# The bittersweet smell of success: Malicious online responses to others achievements

**DOI:** 10.3389/fpsyg.2023.1085317

**Published:** 2023-02-09

**Authors:** Jacob Hornik, Chezy Ofir, Matti Rachamim, Ori Grossman

**Affiliations:** ^1^Coller School of Management, Tel-Aviv University, Tel Aviv, Israel; ^2^Hebrew University and Kinneret Academic College, Tel Aviv, Israel; ^3^Graduate School of Business Administration, Bar-Ilan Universit, Ramat Gan, Israel

**Keywords:** gluckschmerz, eWOM, malicious responses, firestorm, aversive feelings

## Abstract

A prominent recurring theme in social comparison is the concept that individuals are not indifferent to the results that others achieve, and typically seek pleasure while avoiding pain. However, in some cases they behave atypically–counter to this principle. The purpose of this research is to investigate one atypical response, namely gluckschmerz–a negative response to information about others’ success (feeling bad at others’ fortunes). To advance objectives, a mixed-mode of two studies were conducted using a combination of primary and secondary analyzes, and qualitative and quantitative methods. Findings reveal that this aversive feeling encourages consumers to share online “positive” information with others but using negative malicious word-of-mouth narratives. They provide compelling evidence supporting the theory that some of the positive commercial information conveyed through electronic media triggers negative word-of mouth in the form of online firestorms driven by the discordant atypical sentiment of gluckschmerz.

## 1. Introduction

“*As Marty entered his recently promoted colleague’s office, he noticed a photograph of his beautiful family in their new vacation home. He casually adjusts his custom suit and bragged about his upcoming board meeting and marketing speech in Davos. On one hand, Marty wanted to feel genuinely happy for him and celebrate his successes. On the other, you hoped he falls into a crevasse in the Alps. While not forgetting to subtly mention to others that ‘He just got the plum assignment because he plays politics.’” (Tanya Menon, HBR April 2010).*

This story illustrates a one of common manifestations of envy–*gluckachmerz*, feelings of displeasure at others’ success. Evidently, people are not always the most noble creatures. Although they should feel happy when an entity gains success, or sad then the entity suffers, they sometimes show discordant, malicious reactions of gluckschmerz. The sudden discharge of large quantities of negative sentiments to positive events usually toward high achievers or perceived rival individuals, brands, products, companies, managers, and celebrities (hereafter, commercial entities). Evidently, any negative sentiment has the potential to become an online firestorm, defined as “the sudden discharge of large quantities of messages containing negative WOM against a person, company, or group in social media network” (Pfeffer et al., 2014, p. 118). For the commercial entity under slander, such electronic offense can become a possible threat to reputation, especially when magnified by traditional media (Herhausen et al., 2019; Trifiletti et al., 2022). Thus, finding ways to detect and respond to negative eWOM (NeWOM) creates a critical social and managerial priority (Talwar et al., 2019). To date, however, management researchers have paid little attention to gluckschmerz. In this paper, we attempt to fill this gap by examining this negative sentiment a topic which scholars have suggested is “fascinating to learn and a challenge to explore.” (Hess, 2018, p. 308). The real value of studying gluckschmerz in the digital landscape may lie in its influence effect on sharing of “positive” information through the conveyance of negative narratives (negative word-of-mouth, NWOM, Hornik et al., 2015; Hornik, 2018; Hornik et al., 2021b). For example, the social media platform Reddit has numerous forums (“subreddits”) in which high achievers are the subject of discussion. To illustrate, the following recent positive online story received over 40 negative online responses: *Alexey Urazov* a *Russian spokesperson announced that “Montenegro, Saint Vincent and the Grenadines have approved Sputnik V as COVID-19 vaccine.”* Negative responses: *“Citizens’ safety was never Putin’s concern”; “Most Westerners will discredit this vaccine”*; *“*… *a vaccine for suicide!” “Attention! The discovery of the Sputnik V vaccine has been criticized by American scientists for unseemly rapid, corner cutting, and an absence of transparency”* (see Web Appendix A for more online stories).

Story: *Despite Huge Cash Piles, Facebook does not pay dividends. How does Mark Zuckerburg find money to pay for his home bills?*

*Mark Zuckerberg earns money from speaking engagements, sitting on corporate boards, and certainly from investments other than Facebook stock.*

*“It seems that some of his wealth comes from manipulating people.”*

*“The billionaire Mr. Zuck has become a public problem that needs public solutions.”*

*“Zuckerberg is a jerk!”*

*“He is a person who runs after glory. He gives priority to growth and profit over his customers.”*

*“Mark Zuckerberg is a bad boy, ///, not savior of the world.”*

*“His behavior is so bad that it is time for him to go”!*

In a “typical” affective situation people are expected to share positive information using positive WOM (e.g., Septianto and Chiew, 2018). However, this is not true of two “atypical” states: gluckschmerz and its inverse, schadenfreude (feelings of pleasure at others’ misfortune). In the present paper, we advance the novel proposition that people sometimes derive an inherently “dark” pleasure from assessing rival entities and sharing their aversive feelings toward them, initiating or participating in online firestorms. Public discourse has always had its share of hostility and incivility, and the present era is no different in this respect. What is different now is that the current century’s vast, interactive media environment has created more opportunities for public debate, and that moments of malevolent content now spread more rapidly and widely than ever before. The aversive response to this atypical sentiment stems principally from the negative attributions ascribed to a protagonist. As Gore Vidal once put it, “Whenever a friend succeeds a little something in me dies.” The real value in studying gluckschmerz may lie in its effect on dissemination of negative information over the electronic media (Hornik, 2018; Massin, 2018). Our work centers on recent anecdotes evidence and scholars suggestions (e.g., Cecconi et al., 2020; Hornik et al., 2021a) that some NeWOM transmitters might be driven by this inherently malicious sentiment, which might account for some of the strong negative rhetoric found in WOM communication.

Thus, the overall objective of this paper is to present gluckschmerz as a driver of NeWOM communications containing malicious narratives. Considering that gluckschmerz sentiments are common “everyday emotions” (Van de Ven, 2018), it is imperative to investigate and understand the role of this discordant sentiment in internet behavior. Understanding the effect of gluckschmerz on NeWOM might offer an additional account to the prevalence of online firestorms in the online media (Hansen et al., 2018; Herhausen et al., 2019; Talwar et al., 2019). Extant research, however, has not investigated the role of this emotion in shaping eWOM communications. We address this gap by arguing and studying the role of this aversive feeling on sharing online “positive” information with others but by using negative malicious WOM narratives.

The article makes three important contributions to the literature. First, as one of the first empirical works to examine gluckschmerz, it may offer new insights not only for internet research, but for other social science disciplines as well. Second, as research on the drivers of WOM is less developed than research on its outcomes (Söderlund and Rosengren, 2007), and as the majority of relevant studies to date have focused on positive WOM (e.g., Shen and Sengupta, 2018; Talwar et al., 2019), the current study extends the investigation of this subject by exploring a neglected possible determinant of online firestorms and adding to the “negativity bias” discussion (e.g., Norris, 2021). Third, even though the effects and process of social sharing of emotions have been explored in conventional media, little is known about social sharing of emotions in the electronic media (e.g., Hornik et al., 2021a). While gluckschmerz has been referred to in the popular press and recently in psychology, it has received modest attention in the social and management literature. This is regrettable, as many social and managerial events might involve a response to a commercial entity’s success that could provoke malicious feelings. The results of a mixed-mode of two studies we conducted using a combination of primary and secondary analyzes, and qualitative and quantitative methods, provide compelling evidence supporting the argument that some of the positive commercial information conveyed through electronic media triggers NWOM in the form of online firestorms driven by the discordant atypical sentiment of gluckschmerz.

## 2. Conceptual background

**Figure 1:**
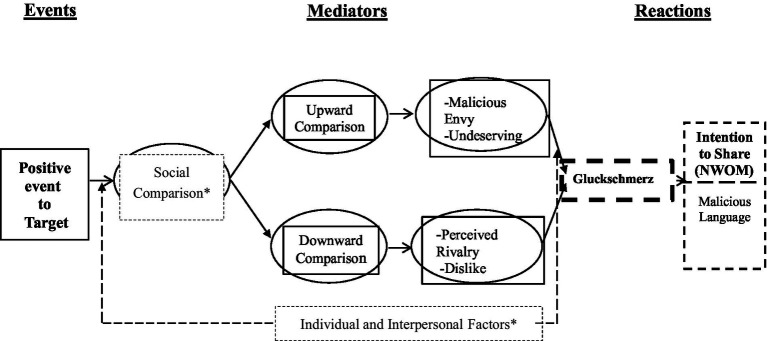
Conceptual framework. *Not treated in this study–for future research.

Our conceptualization merged insights culled from prior studies on gluckschmerz, social-psychology, and the concept of the online firestorm. We propose that this affective state is manifested as an online firestorm usually paired with extreme malevolent and malicious WOM narratives directed at a perceived rival entity because of its arrogance, actions, immorality, or other perceived negative features. Figure 1 outlines our conceptual framework.

### 2.1. Conceptualizing gluckschmerz “your gain, my pain”

Despite its dubious moral reputation, gluckschmerz is indeed a prevalent, fundamental human emotion that reflect the complicated, multidimensional nature of human emotional response. Because comparison with others is a basic, ubiquitous, and potent human proclivity it is usually associated with gluckschmerz (Lange and Boecker, 2019). Humans commonly compare themselves to others as a way of cultivating a positive self-image, self-improvement, and self-motivation. Table 1 summarizes and compares responses to gluckschmerz, as an atypical affect, which scholars (e.g., Smith and van Dijk (2018) have defined as “inherently malicious.” This explains why gluckschmerz is rarely accounted for by frequently used formulations of emotions and, also why it is not among the standard phrases of most languages (van Dijk and Smith, 2019). It seems that there is a broad consensus that gluckschmerz is a perplexing experience (Hess, 2018) leading to wide range of descriptions. For example, Smith and van Dijk (2018) claimed that gluckschmerz is a passive and negative emotion as well as a hateful sentiment. Massin (2018) described it as malicious displeasure, while Gervais and Fessler (2017) regarded it as an “emotional pluripotent.” All these led Johnson (2020) to recently define gluckschmerz as “counterfeit emotion.” This conjecture is captured in the insulting comeback, “Do not hate me because I’m beautiful hate me because I’m young.”

**Table 1:** Response facets in competitive situations.

Typical reactions	Atypical reactions
*Pleasure when another obtains positive outcomes: Freudenfreude (e.g., Chambliss et al., 2012).	**Malicious displeasure when another obtains positive outcomes: Gluckschmerz: (e.g., van Dijk and Smith, 2019).
*Displeasure when another obtains negative outcomes: (e.g., Leach, 2020).	**Malicious Pleasure when another obtains negative outcomes: Schadenfreude (e.g., Hornik et al., 2019).
*Positive affects directed toward underdogs/low achievers (e.g., Feather, 2008).	**Negative affects directed toward underdogs/low achievers (e.g., Feather, 2008).
*Negative affects directed toward top-dogs/high-achievers (e.g., Jin and Huang, 2019).	**Positive affects directed toward top-dogs/high-achievers (e.g., Jin and Huang, 2019).

Similar to gluckschmerz is a concept developed by Feather (2008), which he terms “tall poppy syndrome,” and which refers to the criticism to which successful entities are subjected for their arrogant and attention seeking behaviors. According to Feather (2008), the tall poppy effect arises from both envy and animosity toward entities enjoying great success. Notably, Gluckschmerz has relationships with envy, which involves a negative response to another’s perceived advantage, but unlike envy, gluckschmerz does not require a clear social comparison (Wyer et al., 2019). Based on the anecdotal evidence and conceptual overview we propose the following hypotheses:

*H1*: Participants will express displeasure (gluckschmerz) to the success of an envied and disliked, entity. Research showed that four main factors facilitate the experience of schadenfreude (Smith and van Dijk, 2018):

#### 2.1.1. Malicious envy

As already mentioned, envy is likely to be associated with gluckschmerz. For example, the other entity’s good fortune might provoke the inferiority, associated distress, and any subjective sense of unfairness linked to envy. This will heighten the pain of gluckschmerz, especially, as Roseman and Steele (2018) suggested, any “hopes” that the envied entity might suffer are thwarted by the turnaround of fortunes.

#### 2.1.2. Deservingness

Some studies have revealed that un-deservingness is the leading predictor of the displeasure at others’ fortune (e.g., Hoogland et al., 2015). Research showed that the more fortune was perceived as undeserved, the more it displeases the observer, as it reestablishes a sense of justice and civility (Hess, 2018). Individuals lacking moral qualities evoked higher levels of gluckschmerz because their success was perceived as undeserved. Based on the deservingness concept, gluckschmerz links two important areas of investigations, namely, emotional responses to success and judgments of (un)deservingness that relate to feelings of justice or injustice (Gervais and Fessler, 2017; Smith and van Dijk, 2018).

#### 2.1.3. Dislike

Many instances gluckschmerz follow from prior attitudes or sentiments people have toward a successful entity (Smith and van Dijk, 2018). These are perhaps best understood by whether they like or dislike the entity, for one reason or another. However, it is important to suggest that many cases of gluckschmerz (Hoogland et al., 2015) simply grew from people prior dislikes, regardless to how they might have arisen.

#### 2.1.4. Status

Observing the success of a disliked, or perceived as a rival high achievers, was found to spark more unpleasant feelings compared to observing the success of an regular entity (Feather, 2008; Hornik et al., 2021a). Humans may be primed to constantly develop anti-big-business attitude, and to experience gluckschmerz when they face success. Truly, the tall poppy syndrome suggests that humans feel bad about the success of others who are in positions of high status due to envy and malice.

In sum, gluckschmerz emotions are aggravated by envy and disliked high status entity which its fortune is considered as undeserved. Therefore, we propose the following hypothesis:

*H2*: Gluckschmerz sentiments are mediated by malicious envy and disliking of the rival (involving) entity, as well as a feeling of un-deservingness for the entity’s success.

### 2.2. Emotions—When we care, we share

Emotions are important facets explaining peoples’ behavior. Emotions arise following of an observer’s conscious or unconscious evaluation of some event as positively or negatively relevant to a particular concern or goal (Kwon and Gruzd, 2017). The immediate aftermath of an emotional experience is also characterized by the social sharing process. According to appraisal theory, emotions might have functional consequences as they can motivate humans toward one reaction rather than another. Emotions are composed of two factors: valence and intensity. People tend to assess both sides of the adversity (good vs. bad), this assessment will determine if, and in what intensity they will communicate their emotions to others. NeWOM communication is considered as a personal effort to share information in an unfavorable way online with friends, family and others. As such, transmitting NeWOM messages is a social activity, as individuals share their emotions and opinions experiences with other network members through comments and discussions (Berger and Milkman, 2012).

Venting, the most commonly observed motive in previous research, is consistent with the frequent belief that discussing an emotional experience will reduce its emotional load (Rimé, 2020). In today’s period of anonymous media, people can communicate their messages using forceful, sometimes even violent language, *via* social media. Many forms of negative emotion expression in the electronic media have been studied in the extant literature. For example, trolls intended is to trigger individuals’ inner negative affect, such as fear and anger, resulting in distrust, doubt and irrational reactions (Berger and Milkman, 2012). Recent studies have identified several dimensions that trigger information sharing such as content-related aspects (e.g., hashtag inclusion, topics), people and network characteristics (e.g., rumor, popularity, social capital perception, and homophile) as well as emotions (A recent review see, De Bruyne et al., 2022). This proposes that emotions characterized by increased arousal, such as malicious sadness, anxiety, and amusement and, might boost sharing more than emotions characterized by low arousal, such as distrust or contentment (Lau-Gesk and Meyers-Levy, 2009). Although the system and effects of social sharing of emotions was studied in regular media, less is known about social sharing of emotions in the electronic environments (Kimmel and Kitchen, 2014). This is surprising, since communication in electronic social networks, in the form of talkbacks, blog communities, comments and social media sites, abounds with displays of emotions (Tapanainen et al., 2021; Trifiletti et al., 2022). However, all these may characterize special kinds of people, which Paavola et al. (2016) refer to as “hate holders,” that is, individuals who frequently post deliberately malicious online content (Paavola et al., 2016, p. 104). Hate holders, or what Wang et al. (2019) calls “malicious users,” tend to be dysphoric, tend to focus on negative aspects even in the best of times, and viewing everything through ‘dark colored glasses.’ Thus, emotions play a pivotal role in WOM communication because they relate outer episodes to inner concerns. Therefore, we propose that actively communicating about others’ success provides to some people an emotional outlet well explained by gluckschmerz and expressed through online/social firestorm.

### 2.3. Online firestorms

Commercial entities are increasingly facing enormous online firestorms in response to their arrogance or immoral conduct, and not only from their customers (Hornik et al., 2015; Hornik et al., 2021b; Talwar et al., 2019). Conceptually, online firestorms share elements with rumors which are also carried from person to person, usually by WOM (Pfeffer et al., 2014; Herhausen et al., 2019). Unlike rumors, however, online firestorms might also be based on negative opinions to positive messages. Thus, an online firestorm denotes a phenomenon where the NWOM is intended to insult an entity and is usually without content or convincing evidence (Johnen et al., 2018). The messages in a firestorm are essentially opinions, not fact, and hence have a highly emotional and malicious form (Pfeffer et al., 2014). It has also been suggested that apart from posting messages to express their joy at others’ misfortunes (schadenfreude), individuals might participate in an online firestorm as an outlet to express their negative sentiments, even in response to positive news. Indeed, online firestorms can be triggered by negative but also positive events (e.g., Hansen et al., 2018).

Emotions have a pervasive impact on behavior. Studies on social sharing of emotion show that 90% of affective experiences are carried on to others (see Rimé, 2020). “Talking helps” is a fundamental proposition in clinical psychology, and there is hardly an intervention procedure that does not consider verbalization of feelings to be helpful (e.g., Berger and Milkman, 2012). Although feelings are not verbal features, the verbal use of emotional phrases makes them relatively attainable and contagious. Using affective words in a message practically reveals the underlying intent or basic raw feelings of the sender (Herhausen et al., 2019). Thus, online firestorms seem to be more highly emotional (e.g., “This is frustrating news”). For example, Berger and Milkman (2012) revealed that stories in the New York Times that included more intensive high-arousal emotions (e.g., anxiety, fear, contempt), prompted more hostile email and shared more frequently than stories of low-arousal emotions. Sentiments of this kind were also noted in other contexts. “Negative Double Jeopardy” related to brand hate (Rogers et al., 2017) findings that the most loved brands attract more anti-brand sites, while less loved brands do not have such hate attraction. Similarly, Liao et al. (2020) introduced the concept of “oppositional loyalty” in which inter-consumer brand rivalry and brand community communications are identity-salient events that reinforce the relationship between people-brand identification and influences oppositional loyalty to successful brands. Yip et al. (2018) outlined the possible antecedents of brand hate of “trash-talking” among competing organizations and not only among consumers.

Taken together, harnessing the power of NeWOM requires an understanding of why people talk, and why some things get talked about and shared more than others. The psychology of sharing was acknowledged as a pervasive force shaping schadenfreude and many other behavior phenomena (Hess, 2018; Hornik, 2018; Hornik et al., 2021b). However, missing in most discussions are issues related to counter-empathic sentiments such as gluckschmerz. Evidently, there is something captivating about high achievers. Even the most trivial information about those who are better off can elicit negative sentiments. Indeed, whether it is a fellow employee gaining recognition or a rival brand receiving endorsements, some consumers have experienced moments in which they felt displeasure when an eventuality had positive repercussions for someone else. These sentiments might trigger NeWOM in the form of a malicious online firestorm.

### 2.4. Gluckschmerz emotions as information

Although there are some studies showing that gluckschmerz effects peoples’ emotions, what is not investigated is whether or not those emotions could affect behavior. We propose that gluckschmerz as an aversive emotion may trigger individuals to actively communicating those feelings to other. Gossiping about them, give them “back-handed” compliments. We feel that the real value in studying gluckschmerz may lie in its effect on the dissemination of negative information in the social media. We argue that gluckschmerz sentiments are not only felt privately they may also be communicated to others. Therefore, we propose the following hypotheses:

*H3*: Gluckschmerz sentiments are strongly linked to NWOM and malicious narratives (firestorms).

To test our hypotheses and following the many recommendations (e.g., McKim, 2017), for mixed-methods (qualitative and quantitative) approaches for gaining a deeper insight into a person’s emotions and subjective understanding of events, we start our research using a qualitative study. Thus, using a mixed-method approach, we employed a triangulation process consisting of both quantitative and qualitative research, including both deductive and inductive coding.

## 3. Study 1: Qualitative analysis

Inspired by Berger’s et al. (2020) recent review on the importance of automated textual analysis in marketing research, we adopted the most relevant guidelines and procedures contained therein for Study 1. As a first step in examining differences in affective NWOM content, we applied a qualitative semantic-type data collection method to the study of real stories and their comments sections in the electronic media concerning commercial entities’ episodes of (mis)fortune. This method provided us with a unique opportunity to compute not only the content and narratives (H1 and H2), but also the replication, longevity, and modification (assimilation) of NWOM information.

### 3.1. Procedure

In Study 1, we applied qualitative content analysis to Reddit.com, an increasingly popular news aggregation and discussion website, which is organized into diverse topics, or “subreddits” (Nascimento et al., 2018). Our intention was to select about 80 top-ranked articles on commercial topics, which could be classified as positive stories. We used Reddit’s official API (Reddit, 2020; the Python Reddit API Wrapper (PRAW), for data collection purposes, focusing on three subreddits: r/Business, r/Products, and r/Brands. Due to the Covid-19 pandemic, we were forced to conduct two waves of data collection. Ultimately, we downloaded 83 top-rated positive posts/stories to the selected subreddits. For example: “McDonald’s pouring new lemonade espresso in Poland”; “Nike: Jordan Jumpman Diamonds is going to be released more widely again”; “Jeff Bezos got $7 billion richer in a single day as Amazon shrugged off the coronavirus recession.”

For each selected story, we coded the title, content, comments, timestamp, and scores (i.e., the difference between up votes and down votes). We ended up with 81 usable stories, and used the longevity scores, which are the cumulative number of days consumers have spent on Reddit (the difference between the last day and account creation date). Similar to Hornik et al. (2019) procedure for evaluating differences in language use, we processed comments using Semantria (sematria.com), an automated sentiment analysis platform, which was specially designed to analyze multiple rows of textual content. Availing ourselves of the trial version which enable to analyze up to 10,000 documents. The results indicated clearly whether a comment contained positive, negative, neutral, or very strong sentiments, with an error rate as low as 0.23 and an F-score as high as.85. Quantitatively, we analyzed the malicious narratives on Reddit.com as the percentage of negative comments posted in response to a single editorial relating to a specific positive news story.

To guide raters, we used an inductive analytic method (Berger et al., 2020) to develop a category scheme for the purpose of describing contents characterizing malicious narratives. Categories were drawn from commonly used categories in the literature, most notably, the work of Coe et al. (2014). Over 90% of contents were cod able into the typology (the table in online Web Appendix B provides definitions and examples of each form). After formulating our conceptual definition of online malicious behavior, we operationalized it employing eight categories of malicious communications. This procedure provided the necessary guidelines to extract words and phrases (entity extraction) as well as the relationships between them (Berger et al., 2020). To contend with this issue, two independent coders evaluated the comments first for valence and then for intensity and content assimilation in compliance with the rigorous outlines recommended by Duriau et al. (2007). Coders agreed on *K*(valence) = 0.84; *K*(malicious) = .81of their selections, indicating strong inter-rater reliability. The number of relevant malicious comments were measured by coding every comment section for each of the episodes. Malicious comments were judged to be those that used aggressive and spiteful language, including, among other things, deservedness, malicious envy, and (dis)liking remarks that might offend the corresponding entity. Semantria scores for valence and malicious-type comments were (−) 0.79 and (−) 0.74, respectively, which approximated the coders’ scores.

### 3.2. Results

Table 2 provides descriptive data regarding the commercial-type stories, including karma and longevity scores. Karma indicates how much a poster has contributed to the Reddit community by an approximate expression of the total votes they have gained on their postings (“post karma”) and comments (“comment karma”). When posts get upvoted, that user earns some karma (Nascimento et al., 2018).

**Table 2:** Descriptive data for subreddits in Study 1.

	Positive posts
No. of posts	81
No. of comments	1,741
No. of members	375
Range of no. of comments in posts	1 to 212
Mean no. of comments in posts (SD)	20.9 (12.03)

All 81 positive stories included some (>1) negative comments. 31.2% of the comments were negative indicating a relatively high rate of NeWOM responses to a positive story or gluckschmerz-type responses, supporting H1. Content analysis of the negative responses only clearly revealed that most (73% raters’ scores and (−) 0.80 Semantria index) negative reactions contained malicious-type degrading comments, supporting H1. Although not hypothesized it should be noted that the longevity data revealed that lengthier discussions increased the rate of NWOM, clearly suggesting that online malicious sentiments intensify as discussions grow, a typical feature of online firestorms (Herhausen et al., 2019).

### 3.3. Discussion

Based on the raters’ and Semantria analyzes, Study 1 provided convincing preliminary support for the H1 and H2. Content analyzes of comments posted in response to positive stories on Reddit partially replicated Hornik’s (2018) findings by demonstrating strong malicious sentiments associated with gluckschmerz during NeWOM transmission. Results of Study 1 showed that intensely negative and hostile responses to bittersweet commercial episodes are common in online firestorms, and that some of the malicious narratives were related to gluckschmerz-type sentiments. As suggested by Yi and Oh (2021) using the mixed-methods approach to human emotions and behaviors and in the spirit of triangulation, validating the qualitative data with some quantitative support is recommended. We followed Study 1 with a quantitative study.

## 4. Study 2: Quantitative analysis

The goal of Study 2 was to complement the qualitative data of Study 1 by quantitatively investigating different responses to the gluckschemerz sentiments using a vignette methodology to obtain primary data (Aguinis and Bradley, 2014). The experimental story was a scenario about new owners of Samsung cell phones responding to a sudden success of a perceived rival, namely Apple cell phone. The story was piloted prior to commencing the study to assess gluckschmerz responses to a disliked, envied and undeserving entity, as well as scenario comprehension, and construct validity (Hornik et al., 2021b). Following Terpe’s (2015) suggestion, we used this procedure to also investigate the extent to which the different measures are more or less resistant to context (question order and wording) effects within the survey. The cover story stated that it was a university survey intended to survey opinions on social events (Appendix C provides the scenario and scale items).

### 4.1. Participants and procedure

The study used Qualtrics® online software (version April 2020) and participants recruited *via* the Amazon Mechanical Turk® (MTurk) platform. To reveal potentially small to medium size effects and to add an adequate measure of the interactions between the scenario and the constructs, we decided in advance to recruit approximately 400 American participants, paid for an 8-min task in an online survey. The sample provided an approximately 90% power to reveal a medium main effect of *g* = 0.45 with *α* = 0.05. This sample size was selected with the aim of recruiting at least 100 participants per condition. Missing data were monitored and the cases with missing values less than 5% were substituted by using the mean substitution method. Following recent research (e.g., Arias et al., 2020) on careless responding to online questionnaires including MTurk participants, specifically in studies on sensitive topics involving embarrassing items, such as our study, we used intra-individual response variability as an indicator of flagging participants who showed insufficient efforts, likely providing low-quality data (LQD; e.g., “For system checking please mark response number six”). For the sake of brevity, the various methods and major results are detailed in Web Appendix D. Participants followed an online link that guided them to the Qualtrics® study^1^.They were first presented with an introduction, and once they agreed to join the study, they clicked the START button, which directed them to the task. Participants were promised anonymity and that there were no right or wrong answers. All questionnaires included the following: “To what extent do you agree with the following statements? Please use the following scale where 1 = ‘Strongly disagree’ and 7 = ‘Strongly agree.” We reverse-coded three items to make our questionnaire less prone to socially desirable responses and positive response bias. The questionnaire ended with the following relevant demographics: Age and gender.

### 4.2. Key variables

In addition to the standard gluckschmerz items (Hoogland et al., 2015; Smith and van Dijk, 2018), the questionnaire included the mediating effect items commonly associated with schadenfreude and gluckschmerz (e.g., Lange and Boecker, 2019).

#### 4.2.1. Gluckschmerz

Following Hoogland et al. (2015), gluckschmerz was measured by three statements (e.g., “I’m a little disappointed with Apple’s success”; *α* = 0.87).

#### 4.2.2. Malicious envy

Three items (Hornik et al., 2019; Loureiro et al., 2020); e.g., “When Apple succeed, it makes me feel bad”; *α* = 0.91).

#### 4.2.3. Deservingness

Three items (Feather, 2008; e.g., “Apple did not deserve this,”; *α* =0.82).

#### 4.2.4. Disliking

Two items (Feather, 2008; e.g., “I never liked Apple”; *α* = 0.87).

#### 4.2.5. Personal involvement

Involvement was determined by probing the participants with two questions about whether the event affected them personally or others whom they care about (Garcia et al., 2013; e.g., “I think this information might affect me personally”; *α* = 0.84).

#### 4.2.6. Sharing of information

The dependent variable of intent to communicate and discuss the story *via* NeWOM was based upon a scale developed by Harrison-Walker (2001) and further validated by Goyette et al. (2010). Intent to share the story with others was assessed by a composite score of four behavioral intention questions (i.e., “I will communicate my negative feelings to others”; *α* =0.86). The dependent variable scale appeared directly beneath gluckschmerz and its mediating variable measures (e.g., “I would post my negative opinion while commenting on this Apple information”; *α* = 0.90).

### 4.3. Results

#### 4.3.1. Inter-individual validity measures

To check for possible outliers we conducted Univariate (*via Z*-scores) and multivariate (*via* Mahalanobis Distance and Cook’s Distance) outlier analyzes. The number of subjects participating in this study was 403. Three questionnaires resulted in both a univariate (critically over the *Z*-score of 3.31) and multivariate outlier (beyond the chi-square benchmark of 22.1221 (*p* < 0.001), deviating from the expected univariate and multivariate outlier estimates, which were omitted from the sample. We also excluded three participants who did not mark or missed the attention check for screening out random clicking (i.e., “In this question, we want you to click on number six”), and two participants who did not respond to all the dependent measures. The final sample consisted of 391 participants. Percentages of participants who own Samsung and Apple were 31.6 and 48.4, respectively. This is close to the national market share of the two brands in 2021. There were no main effects or interactions involving the order of question presentations.

*Descriptive statistics* and correlations are displayed in Table 3. First, clear statistical results were found corroborating the influence of gluckschmerz on NeWOM, confirming H3. As predicted, malicious envy, perceived un-deservingness, personal involvement, and disliking were all found to be significantly associated with gluckschmerz. The overall mean for the gluckschmerz condition was 5.21, while the distribution of scores was slightly left/right skewed (Kolmogrov-Smirnov statistic = 0.11, SD = 1.44, skewed = −0.18; and statistic = 0.14, SD = 1.24, skewed = −0.20, respectfully). Second, as expected, a clear statistical difference were found between Samsung and Apple owners in their responses to the scenario. Specifically, applying Hayes’ (2012) template 8 approach to test the differences between the two groups, no effect on malicious sentiment was found among Apple owners (*b* = 0.11, *p* > 0.1; *b* = 0.08, *p* > 0.1). Samsung owners, on the other hand, yielded remarkably high statistical results on the gluckschmerz scales (*b* = 0.43, *p* < 0.05), confirming H1. Participants who claimed either that they did not own a cellphone or that they owned a different brand also exhibited significantly high statistical results on the gluckschmerz measures (*b* = 0.35, *p* < 0.05; *b* = 0.29, *p* < 0.05).

**Table 3:** Descriptive statistics and correlation among Study 2 constructs.

	1	2	3	4	5	6
1. Gluckschmerz	----					
2. Malicious envy	0.69**	----				
3. Undeservingness	0.65**	0.12	----			
4. Disliking	0.62**	0.11	0.08	----		
5. P. Involvement	0.44*	0.06	0.12	0.09	------	
6. Intention to share	0.33*	0.10	0.16	0.18	0.09	-----
Descriptive statistics						
Mean	5.21	5.19	5.23	4.77	4.51	4.52
SD	1.24	1.37	1.51	1.37	1.22	1.34
*α*	0.87	0.91	0.82	0.87	0.84	0.86

**p < 0.01; *p < 0.05; 2-tailed. (1) All measures on a 7-point scale.

#### 4.3.2. Intent to share

A majority of Samsung participants reported a relatively high intent to share the story conveying their negative feelings (*M* = 4.88, SD = 2.21).

#### 4.3.3. Mediation analyzes

To test H2 that malicious envy, perceived deservingness, personal involvement and disliking served as parallel mediators of the effect of a rival positive event on gluckschmerz, a mediation analysis including 5,000 bootstrap resamples and bias-corrected confidence intervals (Preacher and Hayes, 2008) was conducted. It provided an indirect effect of event *via* malicious envy on gluckschmerz, ab = 0.33, SE = 0.10, 95% CI (0.15, 0.57), Sobel *Z* = 4.14, *p* < 0.001. The indirect effects for disliking, ab = 0.52, SE = 0.09, 95% CI (0.33, 0.83), Sobel *Z* = 6.41, *p* < 0.001, deservingness, ab = 0.46, SE = 0.07, 95% CI (0.29, 0.43), Sobel *Z* = 4.11, *p* < 0.001, and personal involvement, ab = 0.36, SE = 0.09, 95% CI (0.25, 0.41), Sobel *Z* = 3.97, *p* < 0.001, were also significant, all in line with H3. Contrasting the central mediators the indirect effect of malicious envy did not differ significantly from the indirect effects of disliking, ab = −0.27, SE = 0.17, 95% CI (0.56, 0.05), deservingness, ab = 0.12, SE = 0.13, 95% CI (0.13, 0.33), and personal involvement ab = 0.36, SE = 0.11, 95% CI (0.27, 0.9), although the latter two did, ab = 0.39, SE = 0.12, 95% CI (0.19, 0.11).

### 4.4. Discussion

Study 2 confirmed H1, H2 and H3 by showing that gluckschmerz sentiments are enhanced and shared (NeWOM) when a high-profile (top-dog) or a leading entity enjoys good fortune, and that dislike, malicious envy, personal involvement, and un-deservingness mediate the propensity for gluckschmerz.

## 5. General discussion

While anecdotal illustrations of the power of rivalry abound, little scrutiny has been made hitherto of the psychological consequences of rivalry. In this research, we provided an initial study of one outcome of perceived rivalry, namely gluckschmerz. In so doing and in the spirit of mixed-methods research in human behavior, two studies were presented: the first supplying qualitative data and the second quantitative data. In Study 1, we found that 31.2% of the comments to the positive stories were negative. Qualitative evidence indicated that most of the negative comments contained intense malicious narratives in the form of firestorms. Study 2, then, complemented Study 1 by supplying quantitative data showing that a large part of the comments on perceived rivals’ success are of the gluckschmerz type embodied in NeWOM. Study 2 also underlined the significant influence of the four mediators of gluckschmerz. Thus, results provided compelling evidence supporting the argument that some of the positive information conveyed through electronic media triggers NWOM in the form of online firestorms driven by the discordant atypical sentiment of gluckschmerz. The findings from the two studies provide novel evidence for extending the range of negativity bias (Norris, 2021) and emotional reactions to others’ (mis)fortune as a predictor of NeWOM.

Some of the findings go hand in hand with prior results. For instance, the correlation between the gluckschmerz and malicious envy found in our research supports Hoogland et al., 2015) findings. Similar to our research, some others work also reported that perceived deservingness, as well as other antecedents, impact gluckschmerz (Hornik, 2018; Hornik et al., 2019; Van Dijk and Smith, 2019). On a macro level, results corroborate prior research addressing the influence of emotions on sharing of information (Rimé, 2020). All adding credence to procedures and findings.

### 5.1. Theoretical significance

The current paper extends prior research on gluckschmerz by advancing the proposition that consumers derive inherent malicious pleasure, in the form of gluckschmerz, from expressing their emotions of various episodes that they receive from others. This study also adds to a growing body of work exploring how atypical sentiments, other than pure emotions, might influence the dissemination of negative information (Massin, 2018). Our results are the first to demonstrate that in addition to having an affective component, gluckschmerz may also have an adaptively tuned cognitive factor. Also, the study makes important contributions to a growing body of studies on NeWOM communication processes. A significant contribution pertains to research on online firestorms (Herhausen et al., 2019; Talwar et al., 2019).

### 5.2. Practical applications

In light of the desire of companies to better apply electronic platforms, it is important to master viral dissemination dynamics and identify posters and contents that are likely to harm reputations. Managers must realize that in the wake of polarizing opinions, the cyber world is laden with malicious content and hate speech. Such knowledge can be used to improve malicious content prevention services and design strategies to attenuate this pattern of inference. Using available dictionary-based automatic text-mining systems, decision makers might be able to estimate the high-and low-arousal levels of negative posts to anticipate their potential diffusion. The more emotion words a post contains the more it is expected to go viral. As suggested by Balaji et al. (2016), when responding to NWOM communications on the electronic media, managers can either engage in proactive or reactive Webcare interventions to mitigate the adverse effects. Proactive Web care refers to service recovery strategies or interventions posted proactively on social media in response to NeWOM communications. Reactive Web care includes interventions posted following specific negative comments from consumers in their eWOM communication. We contend that a timely response to NeWOM communications, either proactively or reactively, will help resolve gluckschmerz type issues.

This research also suggests that in situations of perceived rivalry and negative sentiments bragging might backfire. In these situations, top performers may hide their exceptional qualities in order to avoid gluckschmerz sentiments and NWOM. This work also suggests that managers are better off using messages that highlight the importance of their customers rather than bragging about their brands or managers “Brag with caution.” Also, anticipate a backlash–understanding that envy is a powerful motivator, many managers pit their salespeople against one another for performance rewards. When setting up such competition they should factor in the possibility that gluckschmerz toward winners could lead to later problems. However, in some cases enhancing gluckschmerz sentiments might be used for managerial purposes. For example, the sports network ESPN has advertised its College Football Game schedule with the headline “Watch the team you love and the team you love to hate!” *Sports Illustrated* journal frequently uses negative emotions in its sports editorials and, at times, tries to provoke negative fan feelings by negative editorials about “Most rootable” teams. Also, as prior work in persuasion and suggests. For example, two-sided messages, such as ones that reveal both positive and negative information about a brand, compared to only positive information, increase evaluations of the brand (e.g., Eisend, 2007).

In a society where political candidates’ careers are made or broken by the stories spread about them gluckschmerz may have extensive power to shape the political field. Indeed, disrupting positive information about political candidates with negative narratives proved to be of a balancing value (Shapiro and Rieger, 1992). The theoretical and ethical issues related to gluckschmerz and NeWOM also have implications for educators. For example, results suggest that to explore programs designed to prevent traditional bullying to help prevent online firestorms, like the German Medienhelden (Media Heroes) school educational program (Chaux et al., 2016; Schultze-Krumbholz et al., 2019). Media Heroes seeks to prevent firestorms or cyberbullying mainly by promoting empathy, providing knowledge about definitions, legal consequences, Internet risks and safety, and promoting assertive ways for bystanders to intervene.

### 5.3. Caveats, limitations, and further research

Although our research widens the knowledge on the new determinant of NeWOM communication, it is associated with some limitations, and viable ideas for further research should be identified. First, while hypothetical scenarios are frequently used as research procedures they have several drawbacks. When examining sentiments of a less socially suitable response such as gluckschmerz, the scenario approach may suffer from demand characteristics, which are liable to obscure possible links between gluckschmerz and outcomes. In ensuring convergent validity of our conceptual framework and results, future research needs to replicate our findings when the malicious responses are calibrated by for example, physiological tests, or implicit measures (e.g., affective misattribution concepts). Second, gluckschmerz is a social phenomenon. Therefore, as depicted in Figure 1, future research should consider constructs aiming to explain malicious conduct invasion in a wider social context. On that issue, the Social-Ecological Model (Bronfenbrenner, 1979) can be offer a possible theoretical framework which may have interesting applications in the formalization of malicious content perpetration. Third, as suggested in Figure 1, some personality trait measures might also explain gluckschmerz sentiments. Future work might consider using trait constructs, like self-enhancement and dark triads, to delineate the underlying personalities of this malicious sentiment. Fourth, an important set of constructs likely to impact gluckschmerz are the individuals” cultural background, like their independent versus interdependent self-construal. Fifth, future research should examine the interaction between different communication media (Hornik, 2018) by, for example, exploring how the dynamics of the firestorm change as the negative sentiment shifts from a social media (e.g., Twitter) to a different media. Sixth, NeWOM was our primary dependent variable. Other relevant dependent variables might comprise recall, number of clicks, liking, and purchase intention. Finally, our empirical studies are based on verbal sharing of emotion. There are other, perhaps more immediate ways of communicating emotion, such as facial behavior or posture, which are means evolved to do just that for humans (Johnson, 2020). All these suggestions as well as many other avenues for future research would further expand our understandings on how to manage reputation in the face of gluckschmerz sentiments and online firestorms.

## 6. Summary

Human behavior cannot be fully understood without also studying atypical human sentiment, attitudes, and behavior in prevalent conditions of “bitter joys and sweet sorrows.” We have demonstrated in this paper that when it comes to NeWOM, gluckschmerz sentiments often have a significant role in the dissemination of negative information, and may help us to better understand phenomena such as firestorms, namely the sudden discharge of large quantities of NWOM. Thus, when NWOM circulates, marketers should remember that, “good news travels fast, but bad news travels faster.”

## Data availability statement

The original contributions presented in the study are included in the article/Supplementary material, further inquiries can be directed to the corresponding author/s.

## Ethics statement

Ethical review and approval was not required for the study on human participants in accordance with the local legislation and institutional requirements. The patients/participants provided their written informed consent to participate in this study.

## Author contributions

JH, MR, and OG: literature review, conceptualization, data collection, analyzes, and conclusions. All authors contributed to the article and approved the submitted version.

## Conflict of interest

The authors declare that the research was conducted in the absence of any commercial or financial relationships that could be construed as a potential conflict of interest.

## Publisher’s note

All claims expressed in this article are solely those of the authors and do not necessarily represent those of their affiliated organizations, or those of the publisher, the editors and the reviewers. Any product that may be evaluated in this article, or claim that may be made by its manufacturer, is not guaranteed or endorsed by the publisher.

## Supplementary material

The Supplementary material for this article can be found online at: https://www.frontiersin.org/articles/10.3389/fpsyg.2023.1085317/full#supplementary-material

## Footnotes

^1^ https://biusocialsciences.eu.qualtrics.com/jfe/form/SV3t9Bt8zMQbok2Gx
