# Supplementary material for: The bittersweet smell of success: Malicious online responses to others achievements
Source: Front Psychol. 2023 Feb 9;14:1085317. doi: 10.3389/fpsyg.2023.1085317 (PMC9948090; doi:10.3389/fpsyg.2023.1085317)
Supplement: Supplementary file 1 [file Data_Sheet_1.docx]

**WEB APPENDIXES**

**Web Appendix A:**

**Wal-Mart effect**: S and G are well exemplified by the *"Wal-Mart Effect*," the phenomenon of consumers disliking Wal-Mart, while simultaneously patronizing it. In the Wal-Mart Effect, Charles Fishman (2006)* provides an explanation of how Wal-Mart became one of the largest companies in history. He argues that the giant retailer has amassed such power that it influences everyone's lives, whether they shop at or do business with Wal-Mart or not. Why do consumers dislike Wal-Mart, and yet admit to shopping there? Fishman finds the love–hate relationship consumers have with Wal-Mart fascinating. The love component refers to consumers’ affinity for lower prices and one stop shopping. The hate component, on the other hand, refers to consumers’ disdain for any large, highly successful commercial entity that threatens the wellbeing of smaller, less successful entities as well as the constant search for the Achilles heel of successful entities. These mixed feelings about the company are frequently manifested when consumers relish the giant retailer experiencing a mishap (Schadenfreude), or anguish when the company prospers (Glückschmerz). In addition, in Forbes’ rating of the top corporate hate web sites, *WalMart-Blows.com* received a high score on the hostility level index of 4 out of 5, quoting consumer posts like: "One thing that brings a smile to my face is that the basic laws of economics state there is a limit to the amount of growth that will be profitable. [Wal-Mart] will grow to such a size that it will not be able to control itself and will collapse under its own weight."

* Fishman, Charles (2006), *The Wal-Mart Effect: How the World's Most Powerful Company Really Works—and How It's Transforming the American Economy*, Penguin Press*.‏*

**Starbucks in France:** The research study found that not all consumers feel brand hate at the same level and so-called haters expressed differing severity of hate towards Starbucks, i.e. cold, warm and hot brand hate. Findings revealed that the antecedents of extreme negative affect are to a certain extent dependent on the intensity of brand hate. Franch people expressed both S and G by expressed concern about the global prominence of the coffee chain as exemplified by Starbucks brand dominance: “It seems, you know like Amazon, that they are taking over […]”; and how this was impacting the local economy, “It doesn’t seem fair that they are killing off the independent

cafés in France […] what chance do they have?”. And also stating that Starbuck reveals the “ugly face of American capitalism”.

Reddit////: “*Do French people hate Starbucks? If so, why?”,* March 19, 2008

**Web Appendix B:**

Table: Operational Definition and Examples of Major Forms of Malicious Comments and Narratives

*Form of malicious narrative Operational Definition Examples*

(Intercoder reliability)

**Vulgarity** (α = .90) Using profanity or language that "I hope that the court

would not be considered proper screw…"

(e.g. pissed, screw) in online

comments.

**Name-calling** (α = .71) Mean-spirited or disparaging Intel "Idiots inside"

directed at a commercial entity.

**Aspersion** (α = .63) Mean-spirited or disparaging "Bayers managers are

words directed at entities plan unable to introduce new

or behavior. technologies///"

.

**Convey contempt** (α = .79) The act of despising the entity. "People despise American

made cars".

**Pejorative for speech** (α =.76) Disparaging remarks about the way "Quit crying over

the entity communicates. spilled milk of …".

**Sarcasm** (α = .72) Satirical or ironic utterance "Every person can designed to inflict pain. better shoes than

Adidas"

**Lying** (α = .74) Stating or implying that an act or " McDonalds served

strategy was disingenuous. meat containing

worms"

**False accusations** (α = .91) A charge of fault or offense. "All managers are

corrupted".

**Web Appendix** **C**

**Q4: This is a university questionnaire about people’s feelings concerning smartphones.**

The questionnaire is designed to measure what you are thinking at this moment. The questionnaire is designed to measure what you think this moment. There are, of course, no right answers. The best answer is what you feel is true of yourself presently. Be sure to answer all of the items, even if you are uncertain of the best answer. Do not spend too much time on any one statement. Circle the number that seems to best describe you using the following scale:

1----------2----------3----------4----------5----------6----------7

Strongly Disagree Somewhat Neutral Somewhat Agree Strongly agree

Disagree Disagree Agree

**Please read the scenarios below, and respond, as indicated above, to the statements that follow:**

***Imagine that you own a brand new Samsung cellphone and one of your co-worker is constantly bragging about his Apple cellphone. Suddenly, you were informed that Apple was awarded “cellphone of the year”.***

I’m little disappointed with **Apple** success. 1--2--3---4---5---6—7

Frankly, this is information makes me resent **Apple** more. 1--2--3---4---5---6—7

I enjoyed reading what happened to **Apple**. 1--2--3---4---5---6—7

I feel envious of **Apple**. 1--2--3---4---5---6—7

I feel that **Apple** did not deserve this recognition. 1--2--3---4---5---6—7. I would post my negative opinion while commenting on this information. 1--2--3---4---5---6—7 I will share this **Apple** story with others expressing my disappointments. 1--2--3---4---5---6—7 I like **Apple**. 1--2--3---4---5---6—7 I think this **Apple** information might affect me personally. 1--2--3---4---5---6—7 I am connected with this **Apple** information and its consequences. 1--2--3---4---5---6—7 The information about **Apple** is important for me. 1--2--3---4---5---6—7

What brand computer do you own? Samsung__, Apple __, LG ___, Xiaomi___, Huawei___,

Do not use cellphone___, Other____

My age is _______

I am a: Female___, Male___,

For system checking please mark response number six. 1--2--3---4---5---6—7

Thank you,

**Web Appendix D: Measures for low-quality data**

It is clear that data screening can influence a study’s statistical results, and that low-quality data can distort hypothesis testing, research and practice, particularly when it comes to scale construction. It is somewhat surprising that marketing researchers invest considerable effort in measuring data quality and reliability, and yet pay little attention to monitoring participant reliability, which often is more important than other measures. Various direct and unobtrusive screening methods have been introduced in the research methods literature (e.g., DeSimone & Harms, 2018), most of which are low-cost, easy to administer, and simple to measure. Based on our own previous experiences, we strongly recommend that researchers and practitioners incorporate screening methods into their research designs, and before starting data analyses. Accordingly, in this study we employed the four most common screening methods capable of flagging LQD participants. Throughout the study we monitored LQD to detect responses falling into two categories: insufficient effort (e.g., random or invariant) and deceptive (faking good or intentionally dishonest) responses. We make no claim that all forms of LQD are equally egregious or potentially harmful to our research. The following screening methods were used in study 2:

Bogus items: Participants were flagged as potentially providing LQD if they gave illogical responses to bogus items or failed to follow instructions in instructed items.

Fake good**:** Participants who responded in a manner consistent with social desirability or demand characteristics were assumed to be willing to respond to self-reported effort items in similar ways.

Mahalanobis distance: Outliers need to be usually flagged as potential LQD (DeSimone & Harms, 2018). Mahalanobis distance (D) measures the multivariate distance between an individual’s response vector and the average response vector for all participants who took the questionnaire. D values were calculated using the formula $D= \sqrt{\left( \vec{X}_{i}-\vec{\overline{X}} \right)^{T}*{COV}_{XX}^{-1}*\left( \vec{X}_{i}-\vec{\overline{X}} \right)}$where $\left( \vec{X}_{i}-\vec{\overline{X}} \right)$ represents the vector of mean-centered item responses for participant i and ${COV}_{XX}^{-1}$

represents the inverted covariance matrix of all items. Larger deviation from the normative response pattern yields higher D values and is considered a potential indicator of LQD. A single D statistic was computed for each of the participant using all items. The squared value of D follows a chi-square distribution with degrees of freedom equal to the number of items used in the calculation of D. Participants were flagged if their D^2^ value placed them in the highest 5% of the chi-square distribution.

Personal Reliability: Jackson’s (1976) personal reliability coefficient was computed by correlating the average score on even items with the average score on odd items for each subscale of the SAS. Lower scores indicate LQD in the form of response inconsistency. Personal reliability was computed using a within-person correlation between the vector of even response averages and the vector of odd response averages adjusted for double length using the Spearman-Brown measure. Consistent with Jackson (1976), participants were flagged if their corrected personal reliability coefficient did not exceed 0.30.
